# Scoping review and bibliometric analysis of Big Data applications for Medication adherence: an explorative methodological study to enhance consistency in literature

**DOI:** 10.1186/s12913-020-05544-4

**Published:** 2020-07-24

**Authors:** Salvatore Pirri, Valentina Lorenzoni, Giuseppe Turchetti

**Affiliations:** Institute of Management, Scuola Superiore Sant’Anna, Pisa, Italy

**Keywords:** Big data, Medication adherence, Bibliometric analysis, Scoping review

## Abstract

**Background:**

Medication adherence has been studied in different settings, with different approaches, and applying different methodologies. Nevertheless, our knowledge and efficacy are quite limited in terms of measuring and evaluating all the variables and components that affect the management of medication adherence regimes as a complex phenomenon. The study aim is mapping the state-of-the-art of medication adherence measurement and assessment methods applied in chronic conditions. Specifically, we are interested in what methods and assessment procedures are currently used to tackle medication adherence. We explore whether Big Data techniques are adopted to improve decision-making procedures regarding patients’ adherence, and the possible role of digital technologies in supporting interventions for improving patient adherence and avoiding waste or harm.

**Methods:**

A scoping literature review and bibliometric analysis were used. Arksey and O’Malley’s framework was adopted to scope the review process, and a bibliometric analysis was applied to observe the evolution of the scientific literature and identify specific characteristics of the related knowledge domain.

**Results:**

A total of 533 articles were retrieved from the Scopus academic database and selected for the bibliometric analysis. Sixty-one studies were identified and included in the final analysis. The Morisky medication adherence scale (36%) was the most frequently adopted baseline measurement tool, and cardiovascular/hypertension disease, the most investigated illness (38%). Heterogeneous findings emerged from the types of study design and the statistical methodologies used to assess and compare the results.

**Conclusions:**

Our findings reveal a lack of Big Data applications currently deployed to address or measure medication adherence in chronic conditions. Our study proposes a general framework to select the methods, measurements and the corpus of variables in which the treatment regime can be analyzed.

## Background

Medication non-adherence (MnA) in chronic diseases is one of the most complex issues in Public Health. As highlighted by the World Health Organization (WHO) [1] in 2003, MnA and in general a low degree of adherence to treatment regimes as prescribed lead to poor health outcomes [2, 3] and overall increasing health-care costs [4, 5].

Although MnA is one of the most significant public health issues [6], there are still inconsistent healthcare outcomes and methodological measurements [7], and there is no widely-accepted agreement on its definition [8, 9].

A recent proposal [10] defined medication adherence as the *process by which patients take their medication as prescribed*, and identified three quantifiable phases, *intention*, *implementation* and *discontinuation,* as consistent, measurable and quantifiable steps to analyze the adherence-related management [11].

In our definition of MnA we also include *persistence,* i.e. the length of time between *initiation* and the last dose prescribed [10].

Finding a gold standard to measure the rate of non-adherence or simply a common approach to address the problem is difficult [12]. MnA is a complex issue because diseases differ widely and patients and their drug interaction may react differently. The issue is further compounded by differing beliefs [13], social support and socio-economic status. Moreover, all these variables interact at different levels [14] to determine the magnitude of medication adherence [15].

Different approaches, measurement types and methods have thus been used to explore MnA [16], though this remains an unclear and deeply fragmented field of study [17].

Progress in medical technology has created new solutions to tackle complex issues, as well as new tools and policy strategies to deliver better healthcare services [18] and reduce or make more efficient the overall costs for the National Health Service (NHS) [19]. The proliferation of studies and applications on health information technologies have boosted enormously the possibility to create, understand and obtain valuable information from different sources. Data are generated from medical devices, hospital databases, laboratories and the like, but also from insurance claims, smart-phones, social media, and even more from real-time geolocated sources such as wireless technologies, wearables, and GPSs [20].

This has made the concept of Big Data one of the most promising fields of application in the healthcare domain [21]. Big Data analytics [22] refers to a new generation of technologies and architectures designed to extract value from a very large ***volume*** (i.e. petabytes) of a wide ***variety*** of data, by enabling high-***velocity*** streaming data (3 V’s) across a wide range of sources. These three characteristics are the common elements that currently enable data-intensive technology analysis.

The application of such promising technologies has led to the development of new and effective applications in the healthcare sector [23], although most of the research tends to focus on technical issues [24].

Despite the technologies already in use, little has been done to develop methods, techniques or strategies to apply data intensive analysis to investigate medication adherence issues, particularly in terms of providing long-term strategies for medication adherence. In addition, all the *determinants* (factors) that impact medication adherence need to be considered, especially when these factors, and all the related variables, are modifiable risk factors and sources of information. Once these factors and variables have been recognized they can then be analyzed to determine the magnitude of the MnA, or at least to define a specific strategy to tackle it. Although strategies that define and explore types of medication adherence, measurements and barriers, are quite common, what is extremely important is the ability to engage and maintain a patient’s persistence for the entire period of the treatment. The ability to improve patients’ self-management capabilities and to encourage any lifestyle changes (behaviors) entails a deep understanding of the determinants (factors) that drive patients’ adherence. However there are inconsistent findings on the important role played by medication adherence factors, and the dynamics between the factors, methods and type of measurements have been poorly investigated, and some of its key components have been neglected.

This paper presents the scoping review results of the methods and measurements adopted for medication adherence management in chronic disease. We highlight to what extent Big Data techniques have been deployed to improve or detect evidence-based results for medical decision-making.

1In doing so, we used the taxonomy of Big Data techniques in healthcare developed by Alonso et al. [25], to compare whether Big Data techniques are deployed in medication adherence measurements for chronic diseases.

## Method

**Fig. 1:**
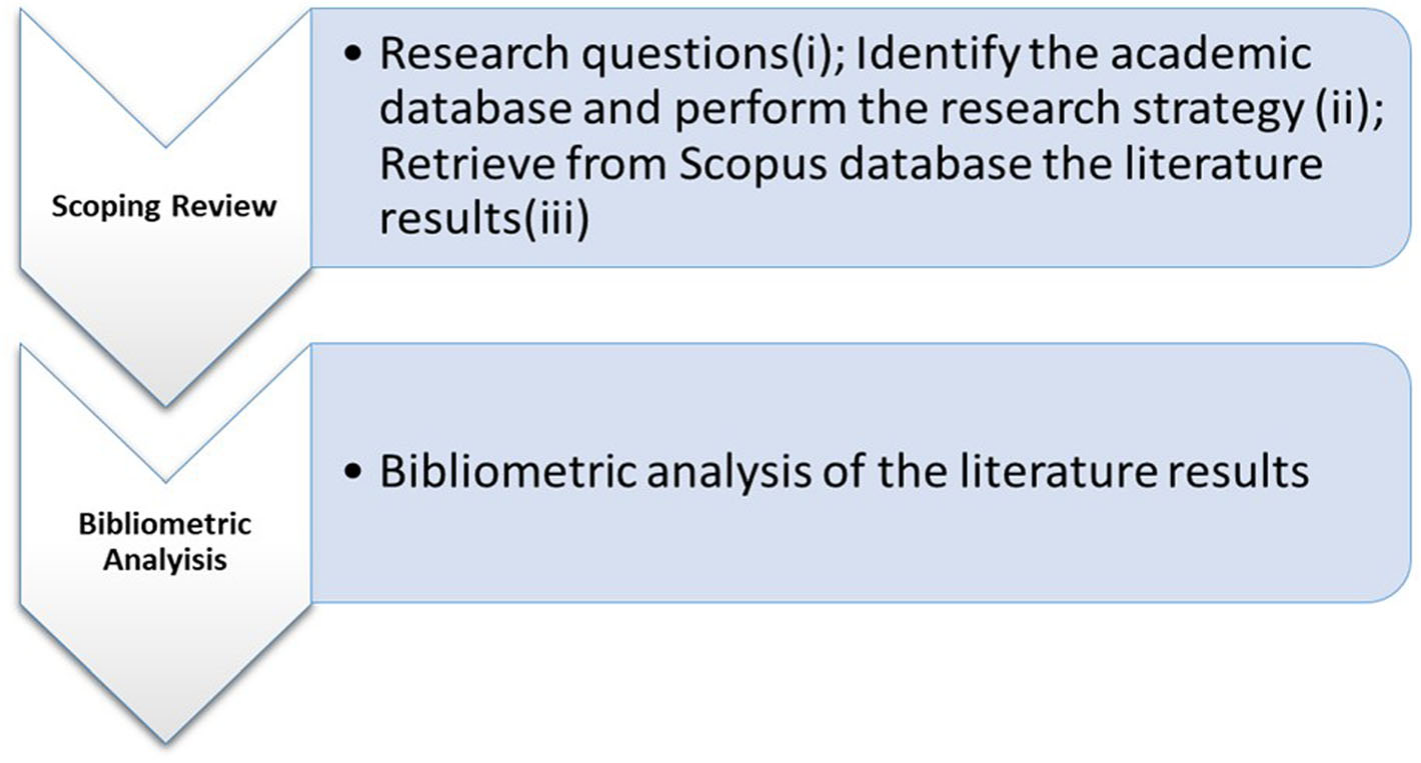
Study process flowchart

We followed the PRISMA Extension for Scoping Reviews (PRISMA-ScR) [26]. We exploited a two steps process to cover all the aspects of our research objectives. See Fig. 1.

*First step*: Perform a Scoping Review adopting the Arksey and O’Malley framework [27].

*Second step*: A bibliometric analysis from the retrieved scoping review results, to analyze the knowledge domains, and possible future research trends.

A scoping review is a relatively new research methodology, particularly effective for summarizing and covering broad research topics, comprising a high number of previous studies, publications, methods, theories, or evidence [28].

Most importantly, a scoping review can pinpoint research gaps without losing research robustness and rigorous quality assessments [29]. Arksey and O’Malley [28] outlined the fundamental five steps for a scoping review: *(1) identify the research question and operationalize the definitions, (2) identify relevant studies through electronic databases and reference lists, (3) establish inclusion–exclusion criteria for the selection of studies, (4) chart the data through a narrative review,11 and (5) analyze, summarize, and report the results.*

To give a more comprehensive and complete overview of the extent and complexity of the topic of medication adherence, we decided to add, as a second step in our research study, a bibliometric mapping analysis [30, 31] using two software programs: VOSviewer [32] and the *bibliometrix* package [33] in R statistical software. We used a bibliometric analysis to measure and visualize the influence of the scoping review results in the scientific community and VOSviewer explore *keyword trends* and the related concepts, as well as *collaboration network maps*.

### Step 1: scoping review

11The aims of this scoping review goals are to: (i) draw up a framework of the types of measurements, study design and methods that were used for medication adherence; (ii) determine whether, and if so which, Big Data techniques lend themselves to improving the decision-making procedures regarding patients’ adherence; (iii) explore the research opportunities and strategies aimed at tackling medication adherence using a data-driven perspective.

#### Stage1.1) development of the research questions

Rq1: What kinds of methods, measurements and approaches are applied to assess medication adherence in chronic diseases?

Rq2: Considering the Big Data techniques used in healthcare [25], are there any data mining methods or techniques used in medication adherence measurement?

Rq3: Are any new data-driven technologies or methods applied in relation to medication adherence?

#### Stage 1.2) framework stage: identification of electronic databases and relevant studies

This study uses articles retrieved from the Scopus electronic database [34], which offers a wider range of journals compared with PubMed and Web of Science [35], and its citation analysis is faster and includes more information [36]. This citation property is a crucial component for our research investigation that influenced many of our decisions regarding data sources.

At this stage, we defined the eligibility criteria (inclusion and exclusion) and defined the searching strategy and keywords used to retrieve the articles. See Table 1.

**Table 1:** Inclusion and exclusion criteria

*Inclusion Criteria*	
- *Articles published in English*	
- *Selected period 01/01/2014–31/12/2018*	
- *Research focused on chronic diseases*	
- *Studies published in peer-review journals*	
- *Type of studies: original articles, reviews, systematic reviews, meta-analyses, scoping reviews, narrative reviews*	
- *Research primarily focused on medication adherence management that aimed to analyze and investigate the relationship between factors/determinants and tools to improve measures or knowledge.*	
*Exclusion Criteria*	
- *Research in which the methods applied and medication adherence measurement are NOT clearly defined*	
- *Research that does NOT take into consideration adherence factors or determinants*	
- *Articles without access to the full text*	
- *Articles that do NOT include relevant information specifically designed to increase or better understand medication adherence*	
- *Research/Articles NOT considering the minimum reporting criteria of EMERGE* [37] *reporting guidelines*	
- *Non adult population included (under 18+ years of age)*	
- *Double citations.*	

All the retrieved articles were exported into *Mendeley*, the software used to organize, select and check the articles.

#### Stage 1.3) screening and selection of publications

We iteratively developed an extensive list of primary and secondary key terms, connected in a Boolean logic and filtering method in order to cover as many research articles as possible linked to the scope of the study. The primary search terms focused on the most common terms in the literature on medication and drug regimes, reflecting the core concept of medication adherence (adherence, compliance and concordance). The secondary type of key terms included a broader set of keywords related to factors, variables, datasets and methodologies applied to obtain specific results on those elements in the literature. A final set of keywords was related to the chronic conditions. The filtering methods included the date range (within the last 5 years), and articles written only in English. See Table 2 for details.

**Table 2:** Search query protocol and flow

*#1*	(“medication adherence” OR “patient compliance” OR “patient persistence”)
*#2*	(“factor*” OR “factors” OR “variabl*” OR “variables” OR “predictor*” OR “predictors” OR “determinant*” OR “database*” OR “dataset*” OR “method*”)
*#3*	(“chronic disease” OR “long term condition*”)
*#4*	#1 AND #2 AND #3
*#5*	(LIMIT-TO (DOCTYPE, “ar “) OR LIMIT-TO (DOCTYPE, “re “))
*#6*	(LIMIT-TO (PUBYEAR, 2019) OR (LIMIT-TO (PUBYEAR, 2018) OR LIMIT-TO (PUBYEAR, 2017) OR LIMIT-TO (PUBYEAR, 2016) OR LIMIT-TO (PUBYEAR, 2015) OR LIMIT-TO (PUBYEAR, 2014))
*#7*	LIMIT-TO (LANGUAGE, “English “)
*#8*	#5 AND #6 AND #7
*#9*	#4 AND #8

## Results

The initial search results from the Scopus database yielded 533 articles. After an initial screening of the titles and abstracts, 285 articles were excluded either because they did not comply with the inclusion criteria and/or because they did not fit in with our study goals. A full-text screening was then conducted on 248 articles and 187 studies were excluded for reasons following the exclusion/inclusion criteria.

**Fig. 2:**
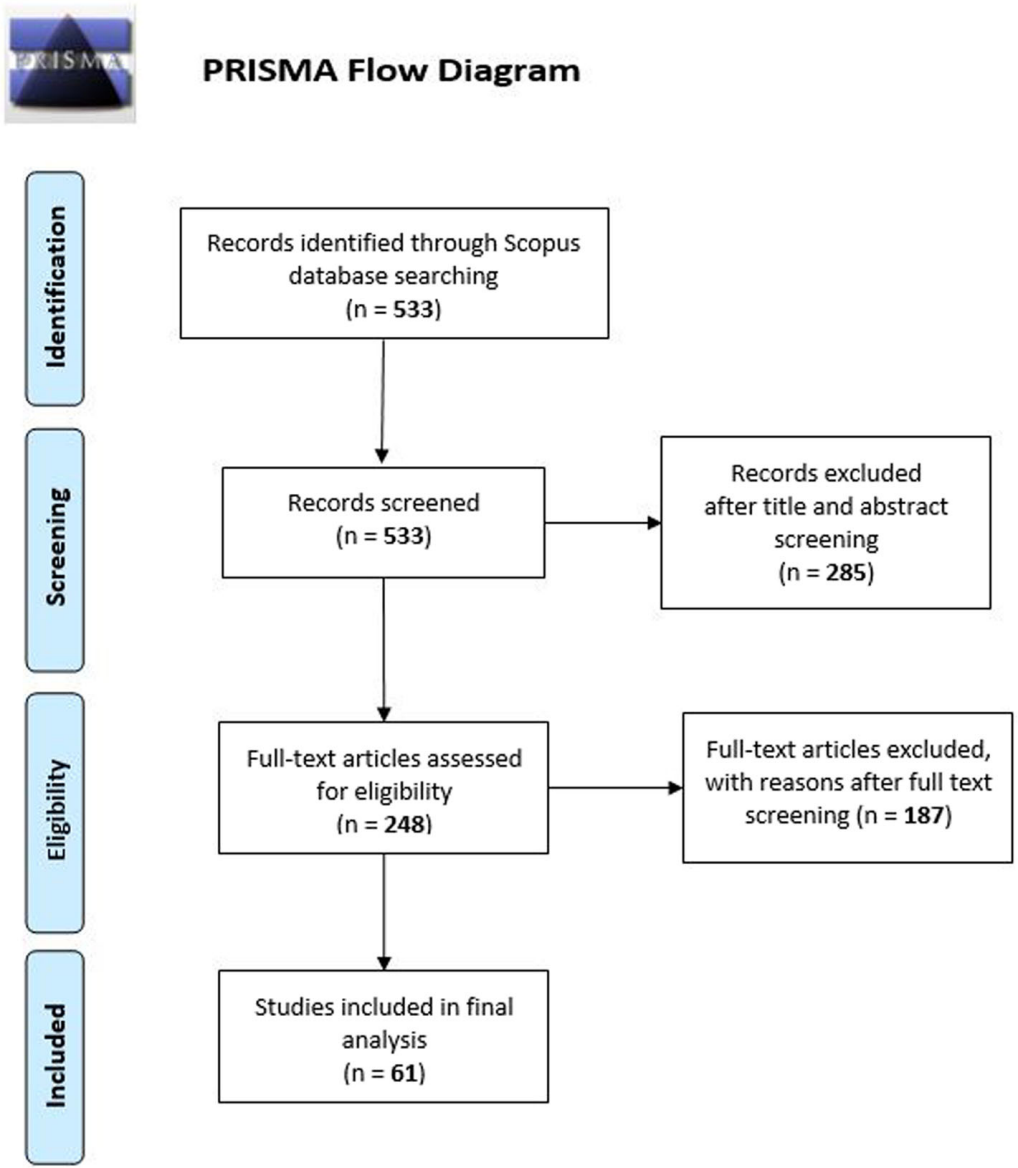
PRISMA flowchart

A total number of 61 articles met all the criteria identified. Using Mendeley, two reviewers first screened the titles and abstracts for eligibility before reading the full texts. In the second part, the reviewers thoroughly examined the full text of all the potentially eligible articles to confirm whether or not they should be included. Disagreement was addressed by consensus after discussion, and a third reviewer was consulted if no consensus was reached. We used the PRISMA flow diagram for the selection flow [38], see Fig. 2.

### Stage 1.4) data charting

In this section and in the following one, according to Arkesy and O’Malley’s Scoping review framework, we present a synthesis and the composition of the 61 articles selected. Although there was a degree of heterogeneity in the literature retrieved, most of the characteristics selected in the inclusion and exclusion criteria enable the results to be categorized in a detailed and homogenous manner. The following Table 3 categorizes the articles based on *authors and title*, *year* of publication, *country* (selected by the first author’s location), main *objective* of the study, *key findings* identified, *methods*, *study duration*, and *population* considered.

**Table 3:** Scoping review results categorized by Arksey and O’Malley’s framework

Authors and Title	Year	Country	Objectives	Key Findings	Methods	Study duration	Population
A. H. A. Al-Ganmi, L. Perry, L. Gholizadeh, and A. M. Alotaibi: ‘Behaviour change interventions to improve medication adherence in patients with cardiac disease: Protocol for a mixed methods study including a pilot randomised controlled trial’.	2018	Australia	The study examine the role of individual, behavioural and environmental factors in predicting medication non-adherence in patients with CVD.	Multi-faceted medication adherence interventions com-prising motivational interviews and text reminders may improve adherence to cardiac medication regimens by targeting individual behaviour change.	Multivariate Logistic Regression	12 months	Adults > 18+
Alsowaida, N., M. Alrasheed, A. Mayet, A. Alsuwaida, M. A. Omair: ‘Medication Adherence, Depression and Disease Activity among Patients with Systemic Lupus Erythematosus’.	2018	Saudi Arabia	Assess the prevalence of both medication non-adherence and depressed mood among Saudi patients with SLE. Validated tools and to explore the impact of both depressive symptoms and disease activity on medication non-adherence.	Medication non-adherence and depressive symptoms are highly correlated prevalent among Saudi SLE patients. Routine screening could facilitate the early detection and management of depression and medication adherence.	Logistic regression	6 months	Adults > 18+
C. Arbuckle et al., ‘Evaluating Factors Impacting Medication Adherence Among Rural, Urban, and Suburban Populations’.	2018	USA	To evaluate differences in prescription medication adherence rates, as well as influencing factors, in rural and urban adults.	The analyses suggest that there is no significant difference in adherence between rural and urban populations; however, the factors contributing to medication adherence may vary based on age and population density.	Multilinear Regression Analysis	NA	Adults > 18+
C. K. Chow et al., ‘TEXT messages to improve MEDication adherence and Secondary prevention (TEXTMEDS) after acute coronary syndrome: A randomised clinical trial protocol’.	2018	Australia	The study aims to investigate whether a cardiac education and support programme sent via mobile phone text message improves medication adherence and risk factor in patients following an acute coronary syndrome	The prevention programme delivered via text messages has the advantages of low cost and being easily automated. These elements can allow these programmes to reach large numbers of people, including those in resource-poor settings and in geographically isolated communities.	Pearson’s chi-squared test (χ2)	6 months and 12 months	Adults > 18+
D. Chandrasekhar et al., ‘Impact of intensified pharmaceutical interventions in medication adherence in chronic kidney disease patients’.	2018	India	The study aims to evaluate the medication adherence behavior of individuals using various cost effective interventional methods.	The result suggests that the periodic counselling by clinical pharmacist at regular intervals can improve the medication adherence by improving awareness and removing the misconceptions about the disease and therapy.	ANOVA and linear regression	12 months	Adults > 18+
G. Martin, N. Patel, Y. Grant, M. Jenkins, R. Gibbs, and C. Bicknell. ‘Antihypertensive medication adherence in chronic type B aortic dissection is an important consideration in the management debate’.	2018	UK	The aims of this study were therefore to identify the rate of antihypertensive medication adherence and predictors of adherence in chronic type B aortic dissection (TBAD).	Medical management remains the mainstay of treatment in uncomplicated TBAD; however, the majority of patients are poorly adherent to their antihypertensive medications.	Linear regression	NA	Adults > 18+
H. Durand et al., ‘Medication adherence for resistant hypertension: Assessing theoretical predictors of adherence using direct and indirect adherence measures’.	2018	Ireland	This study examined theoretical predictors of medication adherence (treatment-related beliefs, coherence of beliefs from experience with medication, habit strength, and pill burden) for patients with hypertension	Associations among unique adherence measures were weak overall, providing further evidence that multiple measures are necessary to accurately assess adherence. Habit strength is a key predictor of adherence for chronic conditions	Hierarchical linear regression	NA	Adults > 18+
H. Kumamaru et al., ‘Using previous medication adherence to predict future adherence’.	2018	USA	To evaluate multiple measures of patient previous medication adherence in light of predicting future statin adherence in a large U.S. administrative claims database.	The study found that previous adherence as a predictor of future adherence was best measured using the PDC. Adding multiple previous adherence measures to the prediction model did not lead to substantive improvement.	C-statistics; concordance statistics	12 months	Adults > 18+
H. Y. Park, S. A. Seo, H. Yoo, and K. Lee. ‘Medication adherence and beliefs about medication in elderly patients living alone with chronic diseases’.	2018	Korea	The aim of this study was to assess medication adherence and its related factors among elderly people living alone with chronic diseases using a conceptual framework	To improve medication adherence of elderly living alone, it is essential to identify barriers to adherence, including their concerns and attitudes toward medications	Multivariable analysis	NA	Adults > 65+
M. Greene, T. Yan, E. Chang, A. Hartry, M. Touya, and M. S. Broder. ‘Medication adherence and discontinuation of long acting injectable versus oral antipsychotics in patients with schizophrenia or bipolar disorder’.	2018	USA	To examine medication adherence and discontinuation in two separate groups of patients with schizophrenia or bipolar disorder	patients with schizophrenia or BD who began receiving long-acting injectable (LAI) antipsychotic had better medication adherence and lower discontinuation risk	Linear and Cox regression models	12 months	Adults > 18+
Monroe, A. K., J. S. Pena, R. D. Moore, K. A. Riekert, M. N. Eakin, S. Kripalani, and others: ‘Randomized Controlled Trial of a Pictorial Aid Intervention for Medication Adherence among HIV-Positive Patients with Comorbid Diabetes or Hypertension’.	2018	USA	A pictorial aid intervention to improve medication adherence for both HIV and common chronic conditions.	Patients with HIV are often medically complex and may have multiple barriers to adherence.Medication adherence is a multifaceted process and adherence promotion interventions require an approach that targets patient-specific barriers.	Logistic regression	6 months	Adults > 18+
O. O. Shiyanbola, E. Unni, Y.-M. Huang, and C. Lanier. ‘The association of health literacy with illness perceptions, medication beliefs, and medication adherence among individuals with type 2 diabetes’.	2018	USA	The study examined the association between health literacy, beliefs in medicines, illness perceptions, and medication adherence in individuals with type 2 diabetes	Health literacy, especially numeracy, needs to be initially addressed before diabetes adherence interventions that address individual concerns about medicines and threatening illness perceptions can work.	ANOVA correlation	NA	Adults > 20+
S. Brandstetter, G. Riedelbeck, M. Steinmann, J. Loss, B. Ehrenstein, and C. Apfelbacher. ‘Depression moderates the associations between beliefs about medicines and medication adherence in patients with rheumatoid arthritis: Cross-sectional study’.	2018	Germany	This study investigate the associations between beliefs about medicines and medication adherence among people with rheumatoid arthritis.	People experiencing more depressive symptoms showed stronger associations between necessity beliefs and adherence as well as attenuated associations between concerns and adherence.	Logistic regression analysis	NA	Adults > 18+
S. Surbhi, I. Graetz, J. Y. Wan, J. Gatwood, and J. E. Bailey. ‘The effect of opioid use and mental illness on chronic disease medication adherence in superutilizers’.	2018	USA	To examine the factors associated with nonadherence to essential chronic medications, with special emphasis on mental illness and use of opioid medications.	This study demonstrated that high levels of opioid medication use are significantly associated with essential chronic disease medication nonadherence among superutilizers. Other risk factors nonadherence were aged < 65 years, low-income status, and a higher number of unique prescribers.	Multivariable associations analysis	6 months	Adults > 18+
V. Menon, N. Selvakumar, S. Kattimani, and C. Andrade. ‘Therapeutic effects of mobile-based text message reminders for medication adherence in bipolar I disorder: Are they maintained after intervention cessation?’.	2018	India	Determine whether text SMS reminders improve medication adherence in patients with bipolar I disorder even after discontinuation of the intervention	The SMS intervention improved medication adherence and attitudes towards medication at the end of the treatment phase. These benefits were maintained for medication adherence but not for attitudes towards medication at the end of the follow-up phase.	(RMANOVAs) Repeated Measures Analysis of Variance	6 months	Adults > 18+
W. Fischer et al., ‘Specific, but not general beliefs about medicines are associated with medication adherence in patients with COPD, but not asthma: Cohort study in a population of people with chronic pulmonary disease’.	2018	Germany	This prospective study investigated the association between beliefs about medicines and medication adherence in patients with asthma and COPD.	Beliefs about medicines are important factors predicting future medication adherence in patients with COPD, but not asthma.	Logistic regression	3 months and 12 months	Adults > 18+
X. C. Tham, H. Xie, C. M. L. Chng, X. Y. Seah, V. Lopez, and P. Klainin-Yobas. ‘Exploring predictors of medication adherence among inpatients with schizophrenia in Singapore’s mental health settings: A non-experimental study’.	2018	Singapore	The aim of this study was to explore the predictors of medication adherence among inpatients with schizophrenia hospitalised at tertiary hospitals in Singapore	this study revealed that there were six factors (insight, religion, side effects, types of antipsychotics, social support from significant others, and nurse-client relationship) which are significant predictive factors of medication adherence	Multivariate Logistic regression	NA	Adults > 18+
X. H. J. Chua, S. Lim, F. P. Lim, Y. N. A. Lim, H.-G. He, and G. G. Teng. ‘Factors influencing medication adherence in patients with gout: A descriptive correlational study’.	2018	Singapore	To examine the factors influencing adherence to urate-lowering therapy in patients with gout in Singapore	Significant differences in medication adherence scores were found among the subgroups of gender, ethnicity, marital status, employment status and presence of comorbidity.	Multiple linear regression	NA	Adults > 18+
Y. Zhang et al., ‘Factors affecting medication adherence in community-managed patients with hypertension based on the principal component analysis: Evidence from Xinjiang, China’.	2018	China	The purpose of this study was to assess the relationship between factors and medication adherence in Xinjiang community-managed patients with hypertension based on the principal component analysis.	Regular medication regimen instruction and better community management services through community-level have the potential to reduce nonadherence.	Binary logistic regression	3 months	Adults > 35+
Y.-M. Huang, O. O. Shiyanbola, and H.-Y. Chan. ‘A path model linking health literacy, medication self-efficacy, medication adherence, and glycemic control’.	2018	USA	The study propose a path model that illustrates the interrelated relationship between health literacy, medication self-efficacy, medication adherence, and hemoglobin A1c (HbA1c).	Medication self-efficacy mediated but did not moderate the relationship between numeracy and diabetes medication adherence.	Bivariate correlations using Spearman’s rho	NA	Adults > 20+
Z. K. Lum, K. Y. K. Tsou, and J. Y. C. Lee. ‘Mediators of medication adherence and glycaemic control and their implications for direct outpatient medical costs: a cross-sectional study’.	2018	Singapore	To investigate the effects of diabetes-related distress and perception of hyperglycaemia on self-reported medication adherence and glycaemic control	Mediation analyses showed a significant indirect effect of diabetes-related distress and perception of hyperglycaemia on medication adherence and HbA1c concentration. People with uncontrolled diabetes were found to incur significantly higher total direct medical costs than those with sub-optimally controlled diabetes.	Linear regression	NA	Adults > 21+
Elsous, A, M Radwan, H Al-Sharif, and A A Mustafa, ‘Medications Adherence and Associated Factors among Patients with Type 2 Diabetes Mellitus in the Gaza Strip, Palestine’.	2017	Iran	Evaluate the adherence to anti-diabetic medications among patients with type 2 diabetes mellitus (DM) seeking medical care in the Gaza Strip, Palestine	Complete adherence to anti-diabetic medications was sub-optimal. New strategies that aim to improve patients’ adherence to their therapies are necessary taking into consideration the influencing factors and the importance of having diabetes educators in the primary care centers.	multiple linear regression	6 months	Adults > 18+
Erku, Daniel A., Asnakew A. Ayele, Abebe B. Mekuria, Sewunet A. Belachew, Bisrat Hailemeskel, Henok G. Tegegn: ‘The Impact of Pharmacist-Led Medication Therapy Management on Medication Adherence in Patients with Type 2 Diabetes Mellitus: A Randomized Controlled Study’.	2017	Ethiopia	Evaluate whether a pharmacist-led medication therapy management could enhance medication adherence in patients with type 2 diabetes.	Pharmacist-led medication therapy management might improve medication adherence and reduce number of hospitalizations in patients with type 2 diabetes.	ANOVA (Analysis of Variance)	6 months	Adults > 18+
Goldstein, Carly M., Emily C. Gathright, John Gunstad, Mary A. Dolansky, Joseph D. Redle, Richard Josephson, and others: ‘Depressive Symptoms Moderate the Relationship between Medication Regimen Complexity and Objectively Measured Medication Adherence in Adults with Heart Failure’.	2017	USA	Assess depressive symptoms as a moderator of regimen complexity in observational study of patients with HF.	Medication regimen complexity in depressive symptoms predicting medication adherence in patients with HF.	Hierarchical multiple linear regression	3 to 6 months	Adults > 18+
Han, Euna, Hyun Soon Sohn, Ju-Yeun Lee, and Sunme Jang, ‘Health Behaviors and Medication Adherence in Elderly Patients’.	2017	South Korea	Explore the relationships of selected health behaviors to medication adherence	Health promotion programs for self-care health behaviors of elderly patientsshould emphasize good medication adherence to achieve successful self-management of diseases.	Multivariate logistic regression	6 months	Elderly adults > 65+
Hayward, K L, P C Valery, J H Martin, A Karmakar, P J Patel, L U Horsfall, and others: ‘Medication Beliefs Predict Medication Adherence in Ambulatory Patients with Decompensated Cirrhosis’.	2017	Australia	Investigate the impact of medication beliefs, illness perceptions and quality of life on medication adherence in people with cirrhosis	Patients having strong concerns or doubting the necessity or helpfulness of their medications should be explored further given the clinical relevance.	ANOVA (Analysis of Variance)	NA	Adults > 18+
Jung, Sun Hoi, Ok Sang Lee, Hyang Sook Kim, Chan Soon Park, Hyun Jung Lee, Kyeng Hee Kwon, and others, ‘Medication Adherence Improvement By Using Administration Timing Simplification Protocol (ATSP) in Cardiovascular Disease Patients’.	2017	South Korea	Evaluate the impact of administration timing simplification protocol (ATSP) on medication adherence and clinical parameters of cardiovascular diseases	Administration Timing Simplification Protocol (ATSP) was shown to be an effective strategy to improve medication adherence in chronic cardiovascular disease patients	statistical significance	3 months	Adults > 18+
Keshishian, Allison, Natalie Boytsov, Russel Burge, Kelly Krohn, Louise Lombard, Xiang Zhang, and others, ‘Examining the Effect of Medication Adherence on Risk of Subsequent Fracture Among Women with a Fragility Fracture in the U.S. Medicare Population’.	2017	USA	The association of osteoporosis medication adherenceand the risk of a subsequent fracture among Medicare-enrolledwomen with a previous fragility fracture.	Enrolled women with low and moderateadherence to osteoporosis medications had a higher risk of a subsequentfracture compared with high adherence patients.	Cox proportional hazards models	NA	Adults > 18+
Kim, Jung-Ae, Eun-Sook Kim, and Eui-Kyung Lee: ‘Evaluation of the Chronic Disease Management Program for Appropriateness of Medication Adherence and Persistence in Hypertension and Type-2 Diabetes Patients in Korea’.	2017	South Korea	Evaluate the effect of CDMP on the appropriateness of medication adherence and persistence in hypertension or type-2 diabetes patients.	Patients visiting the same, single clinic showed a significant increase in appropriate-adherence.	Kaplan–Meier survival analysis	12 months	Adults > 20+
Lau, Ying, Tha Pyai Htun, Kin Sun Chan, and Piyanee Klainin-Yobas: ‘Multidimensional Factors Affecting Medication Adherence among Community-Dwelling Older Adults: A Structural-Equation-Modeling Approach’.	2017	Singapore	Hypothetical model based on the World Health Organization’s five-dimensional model of medication adherence strategy	Finding partially confirmed the conceptual basis of the five-dimensional factors affecting medication adherence.	Structural-equation model (SEM)	NA	Elderly adults > 65+
Lin, C.-Y., M Yaseri, A H Pakpour, D Malm, A Broström, B Fridlund, and others: ‘Can a Multifaceted Intervention Including Motivational Interviewing Improve Medication Adherence, Quality of Life, and Mortality Rates in Older Patients Undergoing Coronary Artery Bypass Surgery? A Multicenter, Randomized Controlled Trial with 18-Month Fo’	2017	Hong Kong	Evaluate the longterm effects of a multifaceted intervention (psycho-education, motivational interviewing, and short message services).	Multifaceted intervention can improve medication adherence in older patients	Multilevel mixed hierarchical models and multiple linear regression	18 months	Adults > 18+
Michetti, Pierre, John Weinman, Ulrich Mrowietz, Josef Smolen, Laurent Peyrin-Biroulet, Edouard Louis, and others, ‘Impact of Treatment-Related Beliefs on Medication Adherence in Immune-Mediated Inflammatory Diseases: Results of the Global ALIGN Study’.	2017	Switzerland	Determine beliefs about systemic medications in patients with immune-mediated inflammatory diseases (IMIDs) and to explore the association of those beliefs and other factors with adherence.	Treatment necessity beliefs were higher than concerns about current medication in patients with IMID. Illness perceptions had a greater impact on treatment necessity beliefs than clinical parameters.	Multivariate logistic regression	11 months	Adults > 18+
Raebel, M A, W Dyer, G A Nichols, G K Goodrich, and J A Schmittdiel, ‘Relationships between Medication Adherence and Cardiovascular Disease Risk Factor Control in Elderly Patients with Diabetes’.	2017	USA	Determine correlates of adherence and examine the effect of meeting Star adherence targetson blood pressure and LDL-C in the Medicare-aged diabetes population.	Adherence to ACEI/ARB is not linked with reduced blood pressure in patients with diabetes who are at least 85 years or with multiple comorbidities.	Poisson regression	NA	Elderly adults > 65+
Tang, K L, H Quan, and D M Rabi, ‘Measuring Medication Adherence in Patients with Incident Hypertension: A Retrospective Cohort Study’.	2017	Canada	Compare adherence rates and associations with mortality using different operational definitions of adherence, and using various methods of handling concurrent medication use.	The range of adherence estimates varies widely depending on the operational definition used. Givenless variation in adherence rates and their stronger association against mortality	Multiple logistic regression models and Cox proportional hazards regressions	12 months	Elderly adults > 65+
Wang, W, G S Chia, I F Tan, S N J Tye, X Wang, B Zhu, and others, ‘Independent Predictors of Medication Adherence among Singaporean Patients Following an Ischaemic Stroke or Transient Ischaemic Attack’.	2017	Singapore	Investigate the independent predictors of medication adherence amongSingaporean patients following an ischaemic stroke or transient ischaemic attack	Nurses play an important role in promoting patients’ medication adherence. Helping stroke patients understand the long-term benefits of their medications is essential to enhance patients’ medication adherence	multiple linear regression	NA	Adults > 18+
Alkatheri, Abdulmalik M., Abdulkareem M. Albekairy, Anan Jarab, Rami Bustami, Nabil Khalidi, Abdulraham Alshaya, and others, ‘Medication Adherence and Treatment Satisfaction among Renal Transplant Recipients’.	2016	Saudi Arabia	Investigate factors that can predict medication adherence and to explore the relationship between treatment satisfaction and medication adherence in renal transplant recipients	Males and RTRs who reported higher treatment satisfaction (convenience and side effects domains) showed better medication adherence	Multivariate logistic regression modelling	12 months	Adults > 18+
Boland, M R S, J F M Van Boven, A L Kruis, N H Chavannes, T Van Der Molen, L M A Goossens, and others, ‘Investigating the Association between Medication Adherence and Health-Related Quality of Life in COPD: Methodological Challenges When Using a Proxy Measure of Adherence’.	2016	Netherlands	Investigate the association between medication adherence and HRQoL, thereby illustratingmethodological challenges	Positive association of adherence and HRQoL was not found, even after adjusting for lifestyle, disease severity, and previous HRQoL.	linear mixed model and Sensitivity analysis	12 and 24 months	Adults > 18+
Garza, Kimberly B., Justin K. Owensby, Kimberly Braxton Lloyd, Elizabeth A. Wood, and Richard A. Hansen, ‘Pilot Study to Test the Effectiveness of Different Financial Incentives to Improve Medication Adherence’.	2016	USA	Measure the relative effectiveness of 2 behavioral economic-based incentive structures to improve medication adherence.	No statistically significant differences in adherence were demonstrated in this small sample of highly adherent participants, larger studies in a more diverse population or with other medications might show otherwise	ANOVA, and Pearson’s correlations	3 months	Adults > 18+
Polsook, Rapin, Yupin Aungsuroch, and Sureeporn Thanasilp, ‘Medication Adherence among Persons with Post-Acute Myocardial Infarction’.	2016	Thailand	The use of multi-stage cluster sampling method involved 348 patients from 9 regional hospitals in Thailand	Findings suggest that nurses should understand that depression, barrier, and self-efficacy are important factors to be considerate to improve medication adherence and improve the quality of life	LISREL (linear structural relations) package used in structural equation modeling (SEM)	December 2011 to February 2013.	Adults > 20+
Tan, C. S.L., G. G. Teng, K. J. Chong, P. P. Cheung, A. Y.N. Lim, H. L. Wee, and others, ‘Utility of the Morisky Medication Adherence Scale in Gout: A Prospective Study’.	2016	Singapore	evaluate the utility of the 8-item Morisky Medication Adherence Scale (MMAS-8) in monitoring medication adherence in a multiethnic Asian gout cohort	MMAS-8 had limited construct validity in assessing medication adherence to ULT in our gout patients	Cronbach’s alpha and intraclass correlation coefficient (ICC)	6 months	Adults > 21+
Beadles, Christopher A., Joel F. Farley, Alan R. Ellis, Jesse C. Lichstein, Joseph P. Morrissey, C. Annette Dubard, and others, ‘Do Medical Homes Increase Medication Adherence for Persons with Multiple Chronic Conditions?’.	2015	USA	Examine the association between medical home enrolment and adherence to newly initiated medications among Medicaid enrollees with MCC	Among Medicaid enrolees with MCC, adherence to new medications is greater for those enrolled in medical homes	Fixed effects regression model and Sensitivity analysis	36 months	Adults > 18+
Freitas, T H, T N Hyphantis, E Andreoulakis, J Quevedo, H L Miranda, G S Alves, and others, ‘Religious Coping and Its Influence on Psychological Distress, Medication Adherence, and Quality of Life in Inflammatory Bowel Disease’.	2015	Brazil	Religious coping plays a role in the adaptation to several chronic diseases. However, the influence of religious coping on IBD-related psychological distress, HRQoL, and treatment adherence remains unknown.	Religious coping is significantly associated with psychological distress, HRQoL, andadherence in IBD.	Hierarchical multiple regression models	NA	Adults > 18+
Kalichman, S C, J Pellowski, C Kegler, C Cherry, and M O Kalichman, ‘Medication Adherence in People Dually Treated for HIV Infection and Mental Health Conditions: Test of the Medications Beliefs Framework’.	2015	USA	Unannounced phone-based pill counts to monitor adherence to psychiatric and antiretroviral medications over a 6-week period	Necessity concerns medication beliefs framework has utility in understanding adherence to multiple medications	Hierarchical linear regression analyses	12 months	Adults > 18+
Kang, C D, P P M Tsang, W T L Li, H H X Wang, K Q L Liu, S M Griffiths, and others, ‘Determinants of Medication Adherence and Blood Pressure Control among Hypertensive Patients in Hong Kong: A Cross-Sectional Study’.	2015	China	Evaluate the determinants of medication adherence and BP control among hypertensive patients in Hong Kong.	Adherence-enhancing interventions should be targeted on younger subjects; employed patients; and those with poor self-perceived health status. Patients who are single and those with comorbiditiesshould be closely monitored for their BP control	Binary logistic regression	6 months	Adults > 18+
Musich, Shirley, Sara Wang, and Kevin Hawkins, ‘The Impact of a Value-Based Insurance Design Plus Health Coaching on Medication Adherence and Medical Spending’.	2015	USA	Evaluate medication adherence, medical services utilization, and combined medical and pharmacy expenditures associated with diabetes and hypertension value-based insurance design VBID) plus health/disease coaching programs implemented by a large employer.	VBID program significantlyreduced pharmacy co-payments for participants and significantlyincreased medication adherence for both participants with diabetes and hypertension while nonparticipants had asignificant medication adherence drop-off.	Propensity score weighting and Regression model	2-year	Adults > 18+
Na, Euihyeon, Seon Jin Yim, Joon Noh Lee, Jung Min Kim, Kyungki Hong, Moon Hwa Hong, and others, ‘Relationships among Medication Adherence, Insight, and Neurocognition in Chronic Schizophrenia’.	2015	South Korea	Identify the association between medication non-adherenceand possible risk factors in a large sample of patientswith chronic schizophrenia.	Identify the relationship between medication adherence, insight, and neurocognition in alarge sample of patients with chronic schizophrenia.	ANOVA and Multiple regression models	1 months	Adults > 55+
Park, Linda G., Jill Howie-Esquivel, Mary A. Whooley, and Kathleen Dracup, ‘Psychosocial Factors and Medication Adherence among Patients with Coronary Heart Disease: A Text Messaging Intervention’.	2015	USA	Compare medication self-efficacy among patients withcoronary heart disease and identify the personal (sociodemographic and clinical characteristics) and psychosocial factors that were associated with.	TM medication reminders and/or health education did not improve medication self-efficacy	ANOVA, Multiple regression analysis	11 months	Adults > 35+
Phillips, L. Alison, Michael A. Diefenbach, Jessica Abrams, and Carol R. Horowitz, ‘Stroke and TIA Survivors’ Cognitive Beliefs and Affective Responses Regarding Treatment and Future Stroke Risk Differentially Predict Medication Adherence and Categorised Stroke Risk’.	2015	USA	Assess the relative importance of four psychological domains – specifically, affective illness, cognitive illness, affective treatment and cognitive treatment for predicting stroke and transient ischemic attack (TIA)	Patients’ cognitive illness beliefs explained the greatest unique variance in baseline and six-month objective categorised stroke risk	Multiple regression model and Pearson bivariate correlation	6 months	Adults > 40+
Rust, Connie F., Cindy Davis, and Matthew R. Moore, ‘Medication Adherence Skills Training for African-American Breast Cancer Survivors: The Effects on Health Literacy, Medication Adherence, and Self-Efficacy’.	2015	USA	Provide information addressing health literacy with respect to medication adherence and self-efficacy in African American breast cancer survivors	A statistically significant relationship was detected between initial health literacy and medication adherence, as well as initial health literacy and self-efficacy	Linear regression model	4–6 months	Adults > 18+
Sandy, Robert, and Ulla Connor, ‘Variation in Medication Adherence across Patient Behavioral Segments: A Multi-Country Study in Hypertension’.	2015	USA	Adherence predictive power of the clusters relative to measures of patients’ concerns over their medication’s cost, side effects, and efficacy.	Predictive power of segments was greater than that for variables measuring concerns over cost, side effects, and efficacy	Cluster segmentation model based on linguistic analysis	NA	Adults > 18+
Seabury, Seth A, Darius N Lakdawalla, J Samantha Dougherty, Jeff Sullivan, and Dana P Goldman, ‘Medication Adherence and Measures of Health Plan Quality.’.	2015	USA	Measure for performance-based reimbursement contractsin healthcare systems.	Plans with higher average adherence had lower rates of disease complications,suggesting that medication adherence measures are potentially useful tools	multivariate regression	NA	Adults > 18 to 65
Shallcross, A J, D A Becker, A Singh, D Friedman, R Jurd, J A French, and others, ‘Psychosocial Factors Associated with Medication Adherence in Ethnically and Socioeconomically Diverse Patients with Epilepsy’.	2015	USA	Psychosocial correlates of medication adherence in a socioeconomically and racially diverse sample of patients with epilepsy.	This study suggest the importance of targeting social support in screening and intervention approaches in order to improve AED adherence among low-income, racially/ethnically diverse patients with epilepsy	Multiple regression model and Pearson bivariate correlation	NA	Adults > 18+
Yue, Zhao, Chen Li, Qi Weilin, and Wang Bin, ‘Application of the Health Belief Model to Improve the Understanding of Antihypertensive Medication Adherence among Chinese Patients’.	2015	China	Insight into antihypertensive medication adherence on the basis of a well-developed behavior theory	The HBM is reliable in predicting medication adherence among Chinese hypertensive patients.	Multivariate logistic regression	NA	Adults > 18+
Abebe, S M, Y Berhane, and A Worku, ‘Barriers to Diabetes Medication Adherence in North West Ethiopia’.	2014	Ethiopia	Assess the magnitude of medication adherenceand factors associated with it among adult persons with diabetes in northwest Ethiopia	Half of the persons with diabetes did not adhere to medications. Adherence was poor among users of traditional treatment and those dissatisfied with services.	Logistic regression	NA	Adults > 18+
Carpenter, Delesha M., Emily A. Elstad, Susan J. Blalock, and Robert F. Devellis, ‘Conflicting Medication Information: Prevalence, Sources, and Relationship to Medication Adherence’.	2014	USA	Explore whether conflicting information is associated with sociodemographic factors, clinical characteristics, and medication adherence	The majority of patients (80.1%) received conflicting information and were most likely to receive conflicting information about medication risks.Physicians, media sources, and the Internet were the most common sources	Pearson correlation and Regression model	NA	Adults > 18+
Hong, J.-S., and H.-C. Kang, ‘Relationship between Continuity of Ambulatory Care and Medication Adherence in Adult Patients with Type 2 Diabetes in Korea: A Longitudinal Analysis’.	2014	South Korea	The relationship between institution-level continuity of ambulatory care and medication adherence in adult patients with type 2 diabetes receiving a new hypoglycemic prescription	Ambulatory care was positively associated with medication adherence, which suggested that a high concentration of ambulatory care visits, whether it’s a physician or an institution, could facilitate delivery of proper medical services to	Random intercept models using a longitudinal technique	NA	Adults > 20+
Iyengar, Reethi N., Dhanur S. Balagere, Rochelle R. Henderson, Abbey L. LeFrancois, Rebecca M. Rabbitt, and Sharon Glave Frazee, ‘Association Between Dispensing Channel and Medication Adherence Among Medicare Beneficiaries Taking Medications to Treat Diabetes, High Blood Pressure, or High Blood Cholesterol’.	2014	USA	Examine the association of pharmacy dispensing channel (home delivery or retail pharmacy) with medication adherence	Where medications are received may impact adherence, even when controlling for PAB. Use of the home delivery dispensing channel may be an effective method to improve adherence for Medicare beneficiaries.	Multiple logistic regression	12 months	Elderly adults > 65+
Langley, Christopher A., and Joseph Bush, ‘The Aston Medication Adherence Study: Mapping the Adherence Patterns of an Inner-City Population’.	2014	UK	Examine nonadherencepatterns to prescribed oral medications within threechronic disease states	The tool has been used to establish nonadherence levels within the three treatment groups and the demographic characteristics indicative of lower adherence levels, which in turn will enable the targeting of interventional support	Correlation	NA	Adults > 18+
Oliveira-Filho, Alfredo D., Donald E. Morisky, Francisco A. Costa, Sara T. Pacheco, Sabrina F. Neves, and Divaldo P. Lyra-Jr, ‘Improving Post-Discharge Medication Adherence in Patients with CVD: A Pilot Randomized Trial’.	2014	Brazil	assess the impact of a low-cost intervention designed to improve medication adherence and clinicaloutcomes in post-discharge patients with CVD	Validated patient self-report instrument for assessing adherence is a potentially effective method to improve adherent behavior and can be successfully used as a tool to guide adherence counselling in the clinical visit.	ANOVA and Cohen’s d effect	12 months	Adults > 55+
Rajpura, Jigar, and Rajesh Nayak, ‘Medication Adherence in a Sample of Elderly Suffering from Hypertension: Evaluating the Influence of Illness Perceptions, Treatment Beliefs, and Illness Burden’.	2014	USA	Collective influence of illness perceptions, medicationsbeliefs, and illness burden on medication adherence of a sample of elderly people suffering from hypertension.	insights into how perceptions of illnessand burden relate to medication adherence in hypertension	Multiple linear regression analysis	NA	Adults > 55+
Xie, Lin, Feride Frech-Tamas, Elizabeth Marrett, and Onur Baser, ‘A Medication Adherence and Persistence Comparison of Hypertensive Patients Treated with Single-, Double- and Triple-Pill Combination Therapy’.	2014	USA	Single-, double-, and triple-pilltreatment regimens among hypertensive patients in a US clinical practice setting.	Pill burden was directly and significantly associated with decreased adherence and persistence with antihypertensive therapies in real-practice settings. Use of fixed-dose combinations that reduce pill burden could help patients to continue treatment and may result in improved clinical outcomes	Logistic regression Cox proportional hazards models	11 months	Adults > 18+

#### Stage 1.5) analyzing data, summarizing and reporting the results

Table 3 reports the key characteristics of the selected articles including important aspects of the study design, and the types of measurements and methods applied and carried out in the articles. This provides a picture of the methods and approaches applied in published papers in order to understand and measure medication adherence in real settings.

Table 4 reports the study design applied, the types of methods used to analyze the variables, the types of measurements used to assess medication adherence, and the list of the major chronic diseases in which they are deployed.

**Table 4:** Characteristics of included Scoping Review studies

Study design	N	Percentage
Cross-sectional study	*24*	*38%*
Randomized Controlled Trial	*13*	*21%*
Retrospective Cohort study	*12*	*20%*
Prospective Cohort study	*5*	*8%*
Longitudinal Observational study	*4*	*7%*
Retrospective claims analysis	*2*	*3%*
Pre and post comparison group	*1*	*2%*
***Diseases***	***N***	***Percentage***
Cardiovascular/Hypertension	*23*	*38%*
Diabetes (Type 1 or 2)	*15*	*25%*
Mental disorder	*6*	*10%*
Others	*4*	*7%*
Osteoarthritis and SLE	*3*	*5%*
(HIV/AIDS)	*2*	*3%*
Stroke	*2*	*3%*
Cancer	*2*	*3%*
Rheumatoid arthritis (RA)	*1*	*2%*
Chronic kidney disease	*1*	*2%*
Gout	*1*	*2%*
Asthma and COPD	*1*	*2%*
***Methods types***	***N***	***Percentage***
Linear regression	*16*	*26%*
Multivariate logistic regression	*16*	*26%*
Analysis of variance (ANOVA)	*8*	*13%*
Statistical significance	*3*	*5%*
Kaplan–Meier survival analysis	*2*	*3%*
SEM (structural equation modelling)	*2*	*3%*
Fixed effects regression model and Sensitivity analysis	*2*	*3%*
Spearman’s rho correlations and Hierarchical linear regression	*2*	*3%*
Cox proportional hazards models	*1*	*2%*
Poisson regression	*1*	*2%*
Intraclass correlation coefficient (ICC)	*1*	*2%*
Cluster segmentation model	*1*	*2%*
Random intercept models	*1*	*2%*
Multinomial regression analysis	*1*	*2%*
Maximum Variation Sampling	*1*	*2%*
Pearson chi-square tests and Sensitivity Analysis	*1*	*2%*
Pearson Correlation rho	*1*	*2%*
Principal component analysis	*1*	*2%*
***Measurement types***	***N***	***Percentage***
Morisky Medication Adherence Scale (MMAS-8)	*22*	*36%*
Proportion of days covered (PCD)	*10*	*16%*
Medication possession ratio (MPR)	*8*	*13%*
Morisky Medication Adherence Scale (MMAS-4)	*6*	*10%*
Medication Adherence Rating Scale (MARS)	*4*	*7%*
medication adherence questionnaire scores (MSSS, MAQ, ARMS, BaMQ, and MASER-R)	*2*	*3%*
Medication pillbox	*1*	*2%*
Electronic caps (Medication Event Monitoring System [MEMS])	*1*	*2%*
Self-Efficacy for Appropriate Medication Use Scale (SEAMS)	*1*	*2%*
Phone-based unannounced pill counts	*1*	*2%*
Medication Adherence Report Scale-5 (MARS-5)	*1*	*2%*
Self-Efficacy for Appropriate Medication Use Scale (SEAMS)	*1*	*2%*
Medication Adherence Self-Report Inventory (MASRI)	*1*	*2%*
Composite scores: Morisky Medication Adherence Scale (MMAS-8) and Medication Adherence Rating Scale (MARS)	*1*	*2%*
Self-designed questionnaire for adherence. China version.	*1*	*2%*

As reported by *Grimes and Schults* [39], a description of clinical study designs can be used to categorize the types of evidence produced. Randomized controlled trials (RCTs) are the most appropriate in determining the causal effects and in reducing the likelihood of potential confounding results.

If rules for RCTs are rigorously applied, the uniform diagnostic criteria for outcomes provide strong statistical and procedural efficacy, making RCTs highly suitable for an evidence-based approach.

RCTs also have weaknesses too, such as organizational complexity, patient recruitment issues, high costs, infrequent external validation, and selection bias [40].

RCTs often do not comprise enough breadth and deep insights commensurate to the complexity of the diseases or to the degree of personalization of treatment needed.

Big Data analytics can fill these knowledge gaps between controlled clinical trials results and clinical practice needs, by collecting data from different sources, and by adopting machine learning techniques able to augment data monitoring and real-world data collection. Big data can provide new insights into disease patterns and help to improve the safety and effectiveness of RCT design [41].

Tapping into these rich resources of real-world data issued through daily clinical practice or collected on a regular basis by hospitals, public bodies or through smart devices, mobile applications, should boost both the output and relevance of controlled clinical research results [42]. However, there are barriers due to regulatory, ethical, and data aspects, as well as the costs of setting up the routine data collection infrastructure [43]. For instance, data management and data linkage might be quite complex to organize and maintain, requiring significant planning and software development. Data accuracy and noisy data are the most challenging tasks to deal with.

Regulatory and ethical aspects pose a major obstacle to the safe exchange and sharing of health records if not well prepared and organized. There are no definitive practical solutions to preserve privacy and to meet the current demand for intensive data-drive solutions. Data discrimination and data breaches are the key factors to avoid when developing a valuable strategy for Big Data analytics implementation in healthcare programs and services [44].

On the other hand, observational methods such as the commonly-used *cross-sectional studies* [45], require less organizational efforts than experimental approaches like RCTs, and provide information on the presence or absence of the exposure in a specific period and act like a time snapshot of the prevalence of an illness in the population under investigation.

It is not the goal of this study to present a list of the best solutions to assess medication adherence in terms of quality or robustness of methods. However, taking into consideration the types of research design that emerged in our scoping review, there is an interesting balance in the study design types between experimental and observational studies. This is quite crucial in understanding adherence phenomenon, because even though RCTs are the gold standard for causal inference, medication adherence is still a patient’s subjective choice and cannot be randomly assigned [46]. Therefore, also observational studies are key to exploring such complex phenomenon.

Looking at the types of models used to analyze and evaluate the effect of the variables on medication adherence, the regression model clearly dominates in the versions of both *linear regression model* (26%), and *multivariate logistic regression* (26%). Choosing the most appropriate model to analyse medication adherence is a critical decision, full of uncertainty and with little consensus regarding a standard method to operationalize this measure [47].

Although our aim is not to judge the quality or the appropriateness of the models used to analyse medication adherence we found that the most used model was the *regression model* whose main purpose is to assess whether or not the independent variable influences the dependent variable [48].

The regression model investigates the relationships between variables as well as the explanatory mechanism underlying the phenomenon under investigation. However, without a strong theory (or model) in which the relationship between variables and determinants is defined, no meaningful decision or result can be made regarding the analyses carried out [49]. Most of the methods highlighted by the results are guided by regression assumptions rather than by a data-driven approach.

Despite the complexity of medication adherence phenomena, we found a lack of studies considering a multivariate approach and time-dependent analyses. For example, latent-group or latent-trajectory analyses and similar methods, sometimes coupled with other methods, seem particularly attractive for studying medication adherence. However, our review found none of these methods in the studies selected.

The capacity of IT analysis in terms of both data storage and processing is immense, however a more effective approach for analyzing data related to medication adherence is needed in order to improve our understanding of indicators or proxies. In particular, techniques and methods to use and to profile adherence behavior over time and among population groups (identified, for instance, by clinical characteristics, socio-demographic data, therapy characteristics) are needed in order to identify potential population risks and behaviors and to establish appropriate methods to assess medication adherence.

### Step 2: the bibliometric analysis

Using R with the package *bibliometrix* [33], and VOSviewer [32], we analyzed 61 articles retrieved from Scopus. Data were analyzed in terms of document statistics, collaboration index, journal impact, country productivity, document citation analysis, and key words. The main goal was to cover the relationships, connections and clusters of scientific production in medication adherence and the use of Big Data.

This kind of analysis can map and identify the hidden connections in a vast bibliography [50] and most importantly, helps to summarize the fragmented research topic of medication adherence.

Below is a series of tables that summarize the descriptive analysis of these data.

As reported in Table 5 there were a total of 360 authors, with 5.9 authors per document. Over a five-year period an collaboration index [51] of 5.9 represents a significant collaboration score that involve the topic of medication adherence.

**Table 5:** Article results main information (2014–2018)

*Main Information on the article data retrieved*	
Articles	61
Sources (Journals)	51
Period	2014–2018
Average citations per article	6.7
Authors	360
Authors per Document	5.9
Co-Authors per Article	6.03
Collaboration Index	5.9

Table 6 summarize the main impact represented by the h index score [52], the journal impact factor [53], and the total citations for the top ten journals and authors. The results presented in this section refer to 2014 to 2018.

**Table 6:** Top 10 journal impact

Source	h_index	Impact factor	Total Citations
Medical Care	158	3.33	38
Patient Education and Counselling	114	2.78	17
International Journal of Cardiology	103	4.03	24
Current Medical Research and Opinion	96	2.57	30
Epilepsy and Behavior	88	2.60	20
Journal of Health Communication	69	1.64	19
Journal of Managed Care Pharmacy	55	2.46	55
Advances in Therapy	51	3.08	16
European Journal of Cardiovascular Nursing	42	2.65	19
Springerplus	26	0.98	19

Almost all of the journals publishing medication adherence-related papers fall into the specific areas of healthcare. No links are evident with the other main research areas covered by journals (e.g., social science, information technology, management, and economic research).

#### Mapping visualization

The 61 articles were visualized using VOSviewer to identify the most important and interesting research areas, aimed at automatically identifying the characteristics and dimensions of the country collaboration map, the document co-citation network, and the keyword trend analysis.

Using clustering techniques [54, 55] the interactions between the selected items can be explored along with how they have shaped the literature, in order to map the scientific knowledge domain and reveal new emerging concepts.

**Fig. 3:**
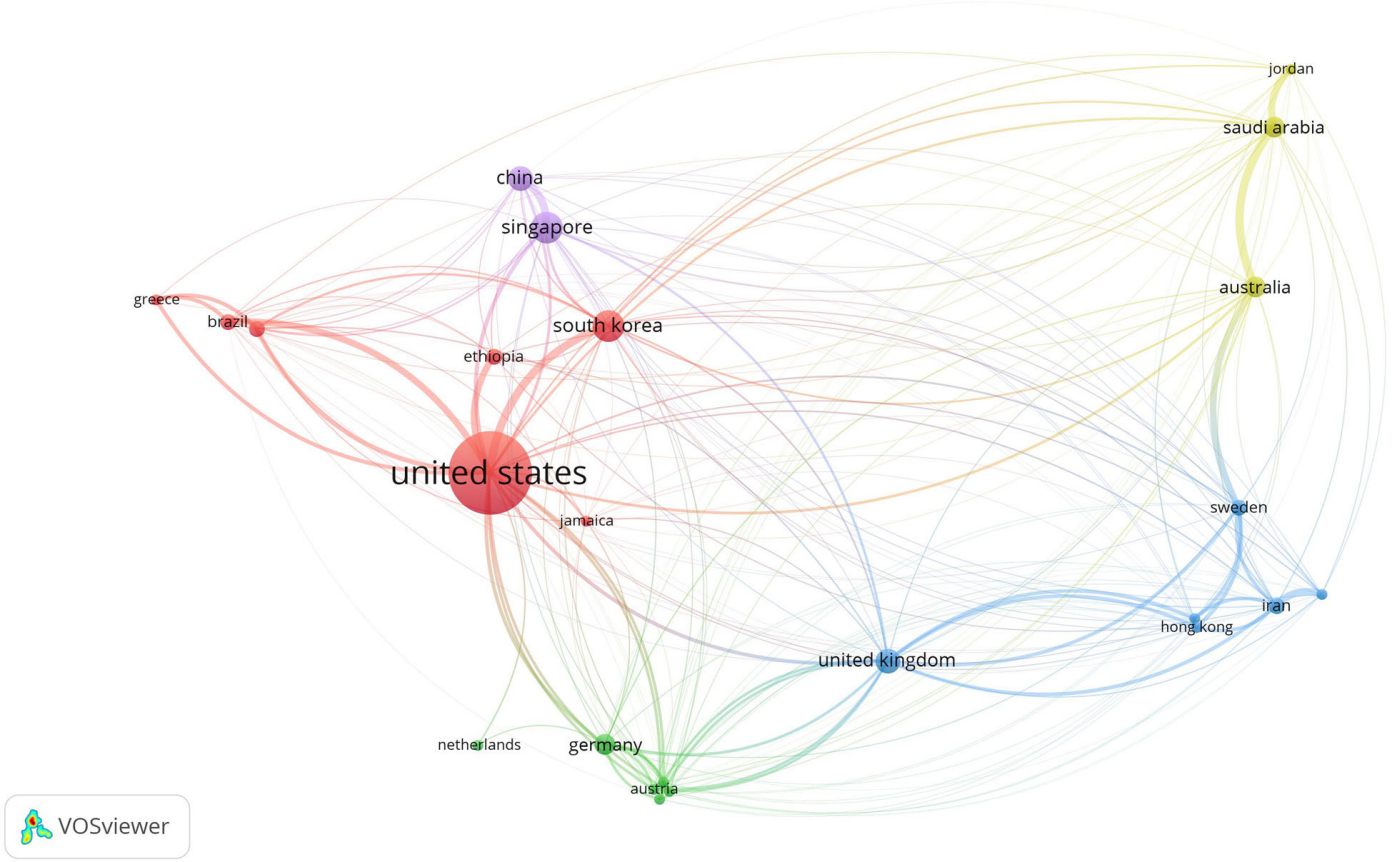
Bibliographic coupling of country collaboration map. The line between two points in the figure indicates that two countries had established a similarity relationship

A total of 24 countries with more than two publications were identified in the 61 articles, see Fig. 3. The five countries with the largest total link strength (TLS) were the USA (TLS = 295.95 and 255 citations), Singapore (TLS = 103.88 and 9 citations), South Korea (TLS = 98.12, 34 citations), China (TLS = 76.00, 40 citations), and the UK (TLS = 116.39 and 22 citations).

The issue of medication adherence is attracting attention globally due to its negative effects on health outcomes, and also due to its negative impact on NHS performance in terms of costs. Clearly, there are differences in socio-economic factors, healthcare systems and specific geographical areas which influence the overall effects of the treatment regime implementation. Assumptions should not be made in terms of quality or by comparing different NHS performances in too much depth. Nevertheless, variables and factors connected to the socio-economic and the healthcare architecture certainly impact greatly on the level of medication adherence. Having a public or a private NHS system, or drug insurance or easy access to treatment, make an important difference in terms of medication adherence rate [56, 57]. For example, *Seth A. Seabury* et al. *(2015)* [58] investigated the association between plan-level measures of health outcomes and medication adherence to assess the viability of adherence as a measure of plan performance, finding that plan-level averages of medication adherence were associated with lower rates of disease-related complications.

Another example is *Reethi N. Iyengar* et al. *(2015)*, who investigated how the dispensing channel impacts on adherence to medications using pharmacy claim data from a large national pharmacy Medicare Part D insurance plan. An enormous database, different sources of information and the related variables-factors specifically associated with the country’s NHS architecture play an important role in the overall dynamics of medication adherence.

Table 7 shows the top 5 documents in terms of citations and link strength in the 61 papers between 2014 and 2018.

**Table 7:** Bibliographic coupling of the top five documents

	Document	Citations	Total link strength
*1*	**Rajpura J.** “Medication Adherence in a Sample of Elderly Suffering from Hypertension: Evaluating the Influence of Illness Perceptions, Treatment Beliefs, and Illness Burden”; (2014).	42	7.00
*2*	**Xie L.** “A medication adherence and persistence comparison of hypertensive patients treated with single-, double- and triple-pill combination therapy”; (2014).	30	8.00
*3*	**Kang C.D.** “Determinants of medication adherence and blood pressure control among hypertensive patients in Hong Kong: A cross-sectional study”; (2015).	24	4.00
*4*	**Shallcross A.J.** “Psychosocial factors associated with medication adherence in ethnically and socioeconomically diverse patients with epilepsy”; (2015).	20	5.00
*5*	**Hong J.S.** “Relationship Between Continuity of Ambulatory Care and Medication Adherence in Adult Patients With Type 2 Diabetes in Korea: A Longitudinal Analysis”; (2014).	20	9.00

**Fig. 4:**
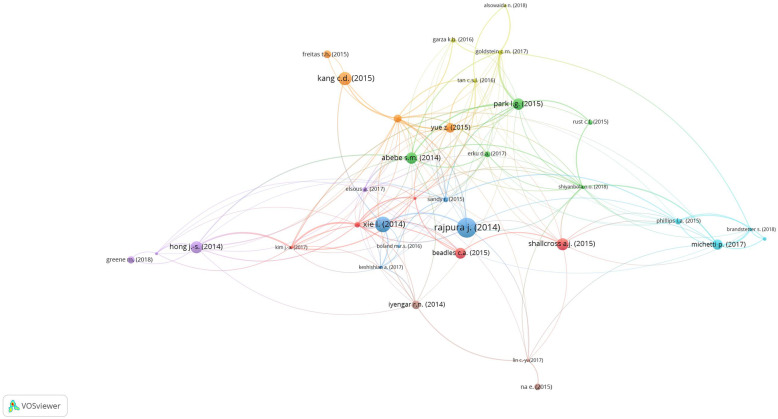
Cluster maps from coupling analysis of bibliographic documents on medication adherence

Bibliographic coupling deploys a similarity measure, using citation analysis to establish a similarity relationship between documents. Bibliographic coupling occurs when two works refer to a common third work in their bibliographies. Bibliographic document coupling involved 36 studies with a minimum of three citations per document. As Fig. 4 shows, eight clusters were identified, with the most cited article being *Rajpura J.* et al [59] (42 citations and TLS = 7.00).

The co-occurrence analysis was adopted to investigate the popular areas and directions of research and was key to monitoring developments in scientific areas and other disciplines. The keywords (used more than 6 times) used in titles and abstracts among the 61 publications were analyzed via VOS viewer to investigate how concepts and topics have evolved.

**Fig. 5:**
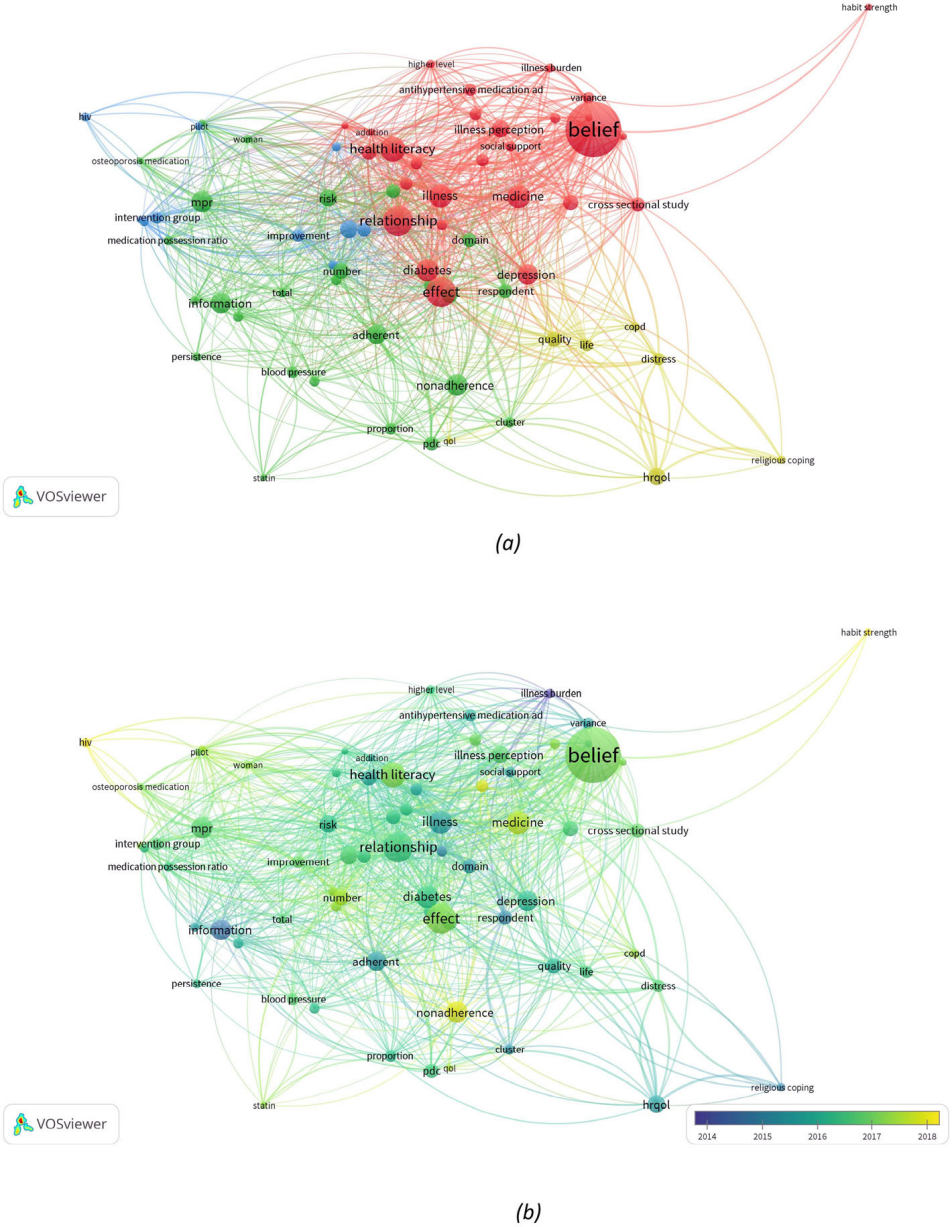
Co-occurrence analysis of the titles and abstracts among the medication adherence studies selected. **a** Mapping and clustering of keywords; three clusters: “Perceptions” (yellow), “interventions” (green and blue), Preferences and needs (red cluster). **b** Mapping and clustering of titles and abstract keywords according to the mean frequency of appearance between 2014 and 2018; keywords in blue appeared earlier (2014) than those in yellow which appeared later (2018)

Figure 5 reports the 71 keywords identified, grouped into approximately three clusters: *“Perceptions”* (yellow), *interventions* (green) and *Preferences and needs* (red cluster) (Fig. 5(a)). In the *perceptions* cluster, the frequently used keywords were *distress, life, health-related quality of life (HRQoL),* and *religious coping*. For *interventions,* the keywords were *non-adherence, risk, medication possession ration (mpr), proportion of day covered (pdc)*. In *Preferences and needs*, which was the largest cluster, keywords were *belief, health literacy, habit strength, illness perception,* and *relationship*.

Co-occurrence clustering of keywords was also analyzed by color view map, based on the mean times they appeared (from 2014 to 2018) in all included publications. Blue indicates that the keywords appeared earlier, and yellow later. As reported in Fig. 5 (b) some keywords such as *adherence* (or *non-adherence*), *medicine*, and *habit strength* underline how the use of these keywords has incorporated the shifting of concepts and definitions. For instance, *adherence* is sometimes used to define the drug regime therapy rather than using *compliance* or *persistence*. Two keywords - *habit strength* and *belief* - underline how important it is to take into consideration the personality and behaviours of the patients in properly managing their drug regime. The size of keywords is proportional to the occurrence rate, and in our case *beliefs*, *relationship* and *effect* were the highest, underlining the importance of a patient’s personality in handling medication adherence.

### Limitations

By design, the scoping review framework does not address specific research questions in a narrow area. In fact, scoping research is meant to address broader exploratory research questions aimed at mapping key concepts, types of evidence, and gaps in the related research domain, selecting and synthesizing existing knowledge, rather than systematically searching and assessing as would take place in a systematic review.

By using just one database (Scopus) as a source of literature, part of the existing knowledge was not included.

Although a rigorous and detailed selection process was adopted, the amount and heterogeneity of literature on the medical treatment regime (e.g. medication adherence, compliance, life-style recommendations) is vast, and identifying common selection criteria is complex. This means that some subjective judgments on the inclusion-exclusion criteria selection were made, thereby obtaining a more comprehensive and homogenous process.

However, bibliometric analysis can contribute to better understanding and investigating a bottleneck in the literature research methodology, which is the difficulty of summarizing in a clear and accessible way the vast amount of literature with common key elements (e.g., subject category, topics, methodology applied) [60].

## Discussion

The results of this scoping review underline how heterogeneous and complex the issue of medication adherence is. A common and robust strategy to tackle this challenge entails devising a more evidence-based and shared approach to improve measurement consistency and appropriate cut-off points to facilitate comparisons among studies [61]. Our review highlighted the lack of a common definition of medication adherence, and the lack of standard method used to measure it. This lack of standardized methods and guidelines impedes on the sharing of good practices and thus the ability to improve the quality of healthcare services, with cost-effective care, and dealing with professional pharmacy services without losing the typical socio-economic organizational characteristics of healthcare entities [62].

Big data enable organizations to analyse massive data sets from a wide range of sources to support evidence-based decision making, through predictive models, and statistical algorithms powered by high-performance systems [63]. Such analyses would enable healthcare organizations to turn information into knowledge by using a combination of existing and new approaches powered by the huge amounts of data generated [64].

However, we found that no specific technology (Big Data) or data-driven solutions are currently in place that offer sufficient accuracy or methodological strength to assess medication adherence.

One of the objectives of this study was to depart from the traditional way of analyzing the literature. We used network analysis techniques to visualize and detect trends and patterns produced in a robust and replicable way. The year-by-year evolution of intellectual and scientific knowledge [65] cannot easily be identified, especially when the number of sources is very high and the concepts so fragmented. We also believe that it is extremely useful to investigate and measure different indicators, such as cluster analyses, keyword trends and other bibliometric measures in order to gather information from unexplored metrics that can offer useful insights for the research topic under investigation.

Our results highlighted that no Big Data (or data mining methods) are currently deployed in medication adherence for chronic conditions, despite its acknowledged beneficial adoption in the healthcare sector. Our bibliometric analysis, and in particular the keyword analysis (Fig. 4), underlined the importance of patients’ preferences, beliefs and habits. These important subjective patient aspects were also deeply investigated by Rajpura J. (2014) [59] and Shallcross AJ. (2015) [66], two of the most-cited documents retrieved. This thus underlines how the influence of illness perception, beliefs and psychosocial factors associated with medication adherence are a major area that can offer further insight supported by powerful Big Data tools.

The literature on medication adherence is widespread and vast, comprising an interdisciplinary approach, and characterized by different research designs and methods for knowledge production. However, it is not easy to obtain a clear overall path of the trends and theoretical approaches in which Big Data analytics can produce rapid and worthwhile results. This is probably because the multifactorial and multivariable aspects that define, make up and influence medication adherence phenomena are still unknown. We believe that developing specific Big Data applications around the patient’s beliefs/preferences would provide valuable insights, new solutions and better clinical feedback.

## Conclusions

We have provided a literature scoping review on the methods, measurements and research design factors affecting medication adherence in chronic disease, exploiting Big Data analysis to improve the clinical decision-making process. We then used the studies selected to develop a literature-driven analysis using a bibliometric methodology to map and identify various future research directions and trends that could provide valuable insights.

Our results show that methods are being implemented to approach medication adherence with Big Data analysis. Embracing a more persuasive policy plan and standardized taxonomy to tackle adherence is needed to make progress in this field, which remains at the forefront of the public health burden.

In addition, the standardized adoption of data knowledge of patients’ beliefs and preferences is needed in order to involve and engage patients in long-term treatment and to understand how their personality impacts on how long they adhere to medical treatment.

Despite the study’s limitations, to the best of our knowledge our scoping review and bibliometric analysis is the first study to combine these two types of methodologies. It thus provides a) a comprehensive understanding of the hotspots and research fronts of medication adherence measurements and methodology assessments; b) a taxonomy of study design, types of measurements, types of methods and variables adopted in the literature retrieved. These could be exploited as a starting point for more precise and tailored evidence-based assessment strategies regarding chronic diseases for medication adherence, which could lead to a more robust application of Big Data analytics.

## Data Availability

Data can be retrieved from Scopus academic database using the presented search query.

## Abbreviations

IT: Information Technology
MnA: Medication non-adherence
NHS: National health Service
PRISMA-ScR: PRISMA Extension for Scoping Reviews
RCT: Randomized Control Trial
WHO: World Health Organization
